# Exercise-Induced Molecular Adaptations in Chronic Non-Communicable Diseases—Narrative Review

**DOI:** 10.3390/ijms262412096

**Published:** 2025-12-16

**Authors:** Héctor Fuentes-Barría, Raúl Aguilera-Eguía, Miguel Alarcón-Rivera, Olga López-Soto, Juan Alberto Aristizabal-Hoyos, Ángel Roco-Videla, Marcela Caviedes-Olmos, Diana Rojas-Gómez

**Affiliations:** 1 Vicerrectoría de Investigación e Innovación, Universidad Arturo Prat, Iquique 1110939, Chile; hefuentes_@unap.cl; 2 Escuela de Odontología, Facultad de Odontología, Universidad Andres Bello, Concepción 3349001, Chile; 3 Departamento de Salud Publica, Facultad de Medicina, Universidad Católica de la Santísima Concepción, Concepción 3349001, Chile; raguilerae@ucsc.cl; 4 Escuela de Ciencias del Deporte y Actividad Física, Facultad de Salud, Universidad Santo Tomas, Talca 3460000, Chile; mrivera3@santotomas.cl; 5 Departamento de Salud Oral, Facultad de Salud, Universidad Autónoma de Manizales, Caldas 170008, Colombia; sonrie@autonoma.edu.co (O.L.-S.); jaristi@autonoma.edu.co (J.A.A.-H.); 6 Dirección de Desarrollo y Postgrados, Universidad Autónoma de Chile, Galvarino Gallardo 1983, Santiago 7500138, Chile; angel.roco@uautonoma.cl; 7 Facultad de Salud y Ciencias Sociales, Universidad de las Américas, Providencia, Santiago 7500975, Chile; 8 Escuela de Nutrición y Dietetica, Facultad de Medicina, Universidad Andres Bello, Santiago 7550000, Chile; diana.rojas@unab.cl

**Keywords:** exercise, myokines, skeletal muscle, intercellular communication, physical exertion

## Abstract

Physical exercise is a potent non-pharmacological strategy for the prevention and management of chronic non-communicable diseases (NCDs), including type 2 diabetes, cardiovascular diseases, obesity, and certain cancers. Growing evidence demonstrates that the benefits of exercise extend beyond its physiological effects and are largely mediated by coordinated molecular and cellular adaptations. This review synthesizes current knowledge on the key mechanisms through which exercise modulates metabolic health, emphasizing intracellular signaling pathways, epigenetic regulation, and myokine-driven inter-organ communication. Exercise induces acute and chronic activation of pathways such as AMPK, PGC-1α, mTOR, MAPKs, and NF-κB, leading to enhanced mitochondrial biogenesis, improved oxidative capacity, refined energy sensing, and reduced inflammation. Additionally, repeated muscle contraction stimulates the release of myokines—including IL-6, irisin, BDNF, FGF21, apelin, and others—that act through endocrine and paracrine routes to regulate glucose and lipid metabolism, insulin secretion, adipose tissue remodeling, neuroplasticity, and systemic inflammatory tone. Epigenetic modifications and exercise-responsive microRNAs further contribute to long-term metabolic reprogramming. Collectively, these molecular adaptations establish exercise as a systemic biological stimulus capable of restoring metabolic homeostasis and counteracting the pathophysiological processes underlying NCDs. Understanding these mechanisms provides a foundation for developing targeted, personalized exercise-based interventions in preventive and therapeutic medicine.

## 1. Introduction

Physical activity is defined as any bodily movement produced by skeletal muscles that requires energy expenditure, whereas exercise refers to a planned, structured, and repetitive form of physical activity aimed at improving or maintaining one or more components of physical fitness [1,2]. In contrast, physical inactivity is characterized by failure to meet the minimum recommendations established by the World Health Organization (WHO), which advise at least 150 min of moderate-intensity aerobic activity or 75 min of vigorous activity per week in adults [3].

Globally, physical inactivity affects approximately one-third of the population, with the highest prevalence reported in the Western Pacific and South-East Asian regions (48.1% and 45.4%, respectively) [4]. This condition is associated with nearly 3.2 million deaths annually [5]. In response, the WHO has set a global target to reduce physical inactivity levels by 15% by the year 2030 [4,6].

Physical inactivity represents one of the most important modifiable risk factors for the development of chronic non-communicable diseases (NCDs), including type 2 diabetes mellitus, cardiovascular diseases, obesity, and several types of cancer [7,8,9,10,11,12]. These conditions impose a substantial global health and economic burden, significantly compromising life expectancy and quality of life [13,14]. Conversely, regular exercise has been consistently associated with reduced incidence and progression of these diseases, along with improvements in metabolic profile, insulin sensitivity, and cardiorespiratory function [15,16].

Over the past decades, growing evidence has unveiled the molecular mechanisms through which exercise exerts its protective and therapeutic effects [17]. Skeletal muscle contraction acts as a potent physiological stimulus that triggers an intricate network of cellular and molecular responses regulating systemic inflammation, energy metabolism, and redox signaling [18,19,20]. Among the most relevant mediators are myokines (such as IL-6, irisin, and myostatin), exercise-regulated microRNAs, epigenetic modifications, and the activation of key intracellular signaling pathways including AMP-activated protein kinase (AMPK), peroxisome proliferator-activated receptor gamma coactivator 1-alpha (PGC-1α), and mechanistic target of rapamycin (mTOR). These pathways collectively modulate mitochondrial biogenesis, fatty acid oxidation, glucose homeostasis, and overall metabolic plasticity [20,21,22].

A comprehensive understanding of these molecular interactions is essential for the development of exercise-based therapeutic strategies targeting NCDs. Within this framework, the present review aims to describe the main molecular effects of physical exercise on chronic non-communicable diseases, emphasizing the underlying biological pathways and their translational potential in personalized and preventive medicine.

## 2. Physiological and Molecular Adaptations to Exercise

Physical exercise triggers a broad range of physiological and molecular adaptations that jointly enhance metabolic efficiency, systemic homeostasis, and cellular resilience [17,23]. At the core of these responses lies skeletal muscle contraction, which operates as an integrative biological stimulus. By combining mechanical tension, neural activation, and metabolic fluctuations, muscle contraction activates interconnected intracellular signaling pathways that coordinate acute and chronic adaptations [24,25,26]. These include rapid metabolic adjustments during exercise and long-term remodeling processes such as changes in gene expression, mitochondrial expansion, and inter-organ communication.

During an acute bout of exercise, muscle fibers undergo marked increases in ATP turnover, cytosolic calcium levels, and reactive oxygen species (ROS) generation [27,28].

It is important to note that these disturbances—normally interpreted as “stress”—are now recognized as essential signals for the activation of molecular adaptation programs [17,23]. For instance, ROS generated during contraction function as second messengers that regulate redox-sensitive transcription factors including NF-κB, Nrf2, and PGC-1α. To maintain a beneficial balance between adaptive ROS signaling and harmful oxidative stress, cells rely on tightly regulated antioxidant systems—including superoxide dismutase, catalase, and glutathione peroxidase—as well as redox-buffering molecules such as glutathione. These mechanisms dynamically adjust ROS levels, ensuring sufficient oxidative cues for signaling while preventing irreversible damage to lipids, proteins, and DNA. Concurrently, the AMP-activated protein kinase (AMPK) detects fluctuations in the AMP/ATP ratio and promotes catabolic processes to restore energy balance [29,30,31]. AMPK activation enhances glucose uptake via GLUT4 translocation and stimulates fatty acid oxidation, collectively fostering an intracellular environment optimized for metabolic homeostasis [32,33,34].

At the transcriptional level, PGC-1α stands out as a master regulator of mitochondrial biogenesis and oxidative metabolism [31]. Its activation through AMPK- and SIRT1-mediated phosphorylation and deacetylation drives the expression of nuclear-encoded mitochondrial proteins and components of the electron transport chain. This coordinated regulation promotes mitochondrial expansion, improved oxidative capacity, and greater metabolic flexibility—hallmarks of exercise-trained skeletal muscle [31,35,36].

Beyond local muscle effects, exercise induces systemic adaptations mediated by the release of bioactive molecules collectively known as exerkines, which include myokines, hepatokines, adipokines, and various metabolites [37,38]. Myokines such as IL-6, irisin, and FGF21 facilitate metabolic crosstalk between muscle and distant organs—including the liver, adipose tissue, pancreas, and brain—modulating glucose and lipid metabolism, insulin action, and inflammatory tone [39,40]. These findings reinforce the concept of skeletal muscle as a dynamic endocrine organ capable of orchestrating whole-body metabolic health [41]. However, the heterogeneity in experimental protocols and analytical methods contributes to inconsistencies—particularly in biomarker quantification (e.g., irisin)—which must be interpreted cautiously.

In addition to these classical pathways, recent evidence indicates that exercise also remodels the intracellular architecture of skeletal muscle fibers, integrating metabolic signaling with organelle-level adaptations. Contractile activity influences mitochondrial dynamics—through coordinated cycles of fission and fusion regulated by AMPK–PGC-1α signaling—and enhances interactions between mitochondria, the sarcoplasmic reticulum, and lipid droplets, optimizing calcium handling, redox balance, and substrate utilization [30,31,35,36]. Key proteins involved in these processes include the fission mediator DRP1, which is activated by exercise-induced phosphorylation, and the fusion regulators MFN1, MFN2, and OPA1, all of which are upregulated or functionally enhanced in response to endurance and high-intensity exercise [42]. These structural adjustments support improved mitochondrial efficiency and reinforce cellular resilience during repeated exercise stimuli.

Chronic exercise training further amplifies these adaptive processes, leading to increases in mitochondrial content, angiogenesis, and insulin signaling efficiency [43]. These long-term effects are supported by cumulative molecular remodeling, including DNA hypomethylation, histone acetylation, and other epigenetic modifications in genes governing oxidative metabolism and inflammation [44,45]. In addition, muscle-enriched microRNAs such as miR-1, miR-133, and miR-206 fine-tune post-transcriptional regulatory networks involved in muscle plasticity, substrate utilization, and recovery [46,47]. While these findings demonstrate the plasticity of skeletal muscle, many epigenetic and miRNA studies rely on small sample sizes or animal models, highlighting the need for more rigorous translational research.

**Figure 1:**
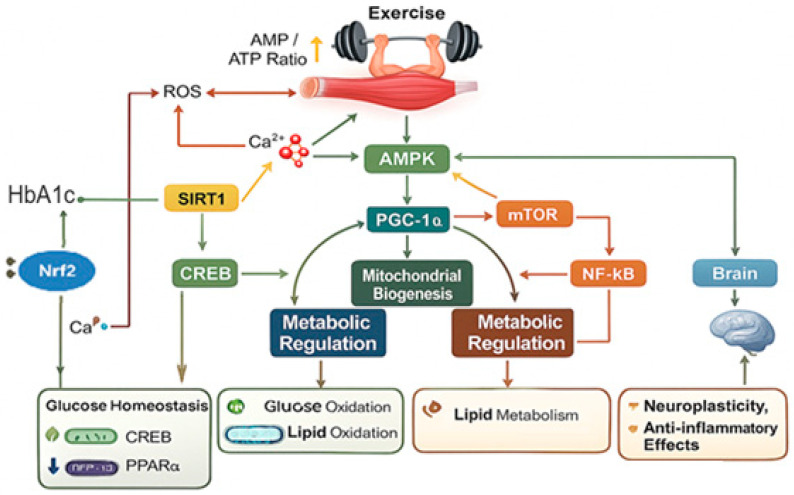
Central Molecular Pathways Activated by Exercise. Source: own elaboration.

Overall, the physiological and molecular responses to exercise represent an integrated biological program that enhances energy sensing, mitochondrial efficiency, and metabolic resilience. These interconnected adaptations not only explain the health benefits of regular physical activity but also establish the mechanistic basis by which exercise prevents and mitigates chronic non-communicable diseases (Figure 1).

## 3. Cellular Signaling Pathways Involved in Exercise Adaptations

Physical exercise activates a complex network of intracellular signaling pathways that coordinate metabolic, structural, and molecular remodeling across multiple tissues, particularly skeletal muscle [1,17]. These signaling cascades underpin the well-documented improvements in energy homeostasis, mitochondrial function, inflammatory balance, and cellular resilience associated with regular physical activity [43]. Among the most thoroughly investigated pathways are AMPK, PGC-1α, mTOR, the MAPK family, and NF-κB. Alongside these canonical axes, exercise-induced epigenetic and post-transcriptional mechanisms, including microRNAs, contribute to the persistence and specificity of long-term adaptations [24,43,48,49].

### 3.1. AMPK Pathway: Energy Sensing and Metabolic Regulation

The AMPK pathway functions as a master metabolic regulator, integrating fluctuations in cellular energy status and orchestrating responses to sustain ATP availability [3,36,50]. During exercise, increased ATP turnover elevates AMP/ATP and ADP/ATP ratios, activating AMPK through phosphorylation at Thr172 by upstream kinases such as LKB1 and CaMKKβ. This activation promotes a shift toward catabolic pathways that generate ATP while suppressing energy-demanding anabolic processes [1,17,48].

In skeletal muscle, AMPK activation enhances glucose uptake through insulin-independent GLUT4 translocation and stimulates fatty acid oxidation via phosphorylation of acetyl-CoA carboxylase (ACC), lowering malonyl-CoA levels and facilitating mitochondrial fatty acid import through CPT1. These coordinated responses maintain ATP supply during repeated contractions [51,52,53,54].

Beyond its acute role, AMPK contributes to long-term metabolic adaptation by activating PGC-1α—either directly or through interactions with SIRT1—thereby promoting mitochondrial biogenesis and oxidative metabolism [30,31,43]. These processes underpin the enhanced metabolic flexibility characteristic of trained skeletal muscle.

AMPK-mediated adaptations extend across tissues. In the liver, AMPK inhibits gluconeogenesis and lipogenesis while promoting fatty acid oxidation [50]; in adipose tissue, it reduces lipogenesis and enhances lipolysis; and in vascular endothelium, it improves nitric oxide bioavailability through eNOS activation [55,56]. Collectively, AMPK serves as a key integrator of metabolic and vascular responses to exercise, with chronic activation protecting against insulin resistance, dyslipidemia, and cardiovascular dysfunction [57].

Critically, although AMPK is frequently portrayed as unequivocally beneficial, its activation is highly context-dependent and varies by exercise intensity, nutrient status, and fiber type—factors often underreported and limiting cross-study comparisons.

### 3.2. PGC-1α Signaling: Master Regulator of Oxidative and Mitochondrial Adaptation

PGC-1α is a central transcriptional coactivator driving the molecular remodeling of skeletal muscle in response to resistance exercise [58]. Activated by both endurance and resistance exercise, PGC-1α regulates mitochondrial biogenesis, oxidative phosphorylation, angiogenesis, and fiber-type transformation toward oxidative phenotypes [30,31,43,51].

Molecularly, PGC-1α integrates convergent signals from AMPK, SIRT1, and p38 MAPK, which modulate its phosphorylation, deacetylation, and nuclear translocation [59]. In the nucleus, it coactivates NRF1, NRF2, and ERRα, enhancing expression of nuclear-encoded mitochondrial genes and components of the electron transport chain [60,61]. PGC-1α also facilitates mitochondrial DNA replication by stimulating TFAM, expanding mitochondrial content and capacity [62,63].

Beyond metabolism, PGC-1α increases antioxidant enzyme expression (e.g., SOD2, catalase), attenuates inflammation by antagonizing NF-κB, and induces VEGF-driven angiogenesis, collectively improving oxidative tolerance and muscle perfusion [64,65,66,67].

Systemically, PGC-1α improves insulin sensitivity, enhances lipid utilization, and reduces ectopic fat accumulation, with emerging evidence pointing to roles in liver and adipose tissue via exerkine-mediated inter-organ communication [43,58,60,63,67].

A critical limitation in the current literature is the tendency to attribute a wide range of adaptations to PGC-1α without distinguishing between isoforms, cell-specific expression, or the contribution of compensatory pathways—issues that remain underexplored in human studies.

### 3.3. mTOR Pathway: Protein Synthesis, Muscle Hypertrophy, and AMPK Interplay

The mTOR regulates cell growth, protein synthesis, and nutrient sensing, particularly in response to mechanical load and amino acid availability [68]. During resistance exercise, activation of mTORC1 promotes muscle hypertrophy and protein accretion [69].

Mechanical signals and amino acids—especially leucine—activate mTORC1 via the Rag GTPases and PI3K/Akt pathway [70]. Activated mTORC1 phosphorylates S6K1 and 4E-BP1, stimulating translation initiation and ribosomal biogenesis [71,72], ultimately driving muscle fiber growth.

A critical aspect of this pathway is its antagonistic interplay with AMPK [48,73]. Under low-energy conditions, AMPK inhibits mTORC1 via Raptor phosphorylation, suppressing anabolism to preserve ATP [74]. This dynamic ensures coordinated metabolic efficiency: mTOR dominates when energy and nutrients are abundant, whereas AMPK prevails during energetic stress.

Beyond muscle, mTOR influences insulin signaling, autophagy, and lipid metabolism [75]. Dysregulation contributes to insulin resistance, obesity, and certain cancers, positioning exercise-mediated modulation of the AMPK–mTOR axis as a key mechanism for systemic metabolic health [76,77].

Despite extensive study, most evidence stems from controlled laboratory settings with limited ecological validity; real-world exercise responses may vary substantially due to nutritional timing, age, and sex-specific differences—factors requiring greater investigation.

### 3.4. MAPK and NF-κB Pathways: Inflammation and Oxidative Stress Regulation

The MAPK family—including ERK1/2, JNK, and p38 MAPK—transduces mechanical, metabolic, and cytokine-derived signals during exercise [78]. ERK1/2 primarily supports growth and differentiation, facilitating structural remodeling after resistance exercise [79]. In contrast, JNK and p38 MAPK respond to metabolic and oxidative stress, linking ROS generation with transcriptional control [80].

p38 MAPK interacts directly with PGC-1α, reinforcing mitochondrial biogenesis and antioxidant defense while regulating cytokine expression [80,81]. These effects illustrate the functional convergence between stress signaling and metabolic adaptation.

NF-κB, a central transcription factor in inflammation and redox regulation, is transiently activated during exercise by ROS and cytokines [66]. Moderate, acute activation induces antioxidant and cytoprotective gene expression, supporting adaptive hormesis [66,82]. In contrast, chronic NF-κB activation—associated with inactivity or overtraining—promotes inflammatory cytokine production and muscle catabolism [83]. Regular exercise mitigates chronic inflammation by enhancing IκB expression and suppressing systemic inflammatory mediators [84].

These pathways exemplify the dual nature of ROS—as essential signals or harmful stressors depending on intensity, duration, and recovery [19,49]. A critical challenge is the lack of standardized methods to quantify real-time redox dynamics in humans, limiting interpretation across studies.

### 3.5. Epigenetic and microRNA Modulation: Post-Transcriptional and Chromatin-Level Control

Exercise exerts powerful regulatory effects on gene expression through epigenetic mechanisms and microRNA-mediated post-transcriptional modulation [85]. These processes serve as a “molecular memory,” translating behavioral cues into persistent genomic adaptations [17,23,86].

Exercise alters DNA methylation in genes associated with mitochondrial function, glucose transport, and lipid metabolism [87,88]. Hypomethylation of promoters for PGC-1α, TFAM, and GLUT4 enhances transcriptional activity and metabolic plasticity [51]. Concurrent histone modifications—including acetylation and methylation—modulate chromatin accessibility, with SIRT1-mediated deacetylation linking energy status to chromatin structure [89,90].

MicroRNAs (miRNAs) fine-tune these adaptations. Muscle-enriched myomiRs (miR-1, miR-133a, miR-206) regulate myogenesis, mitochondrial biogenesis, and muscle regeneration [46,47]. Circulating miRNAs act as systemic messengers mediating communication between muscle and distant organs such as liver, adipose tissue, and brain [90,91].

Emerging findings suggest possible transgenerational effects of exercise-induced epigenetic remodeling, indicating that physical activity may influence metabolic phenotypes beyond the individual [92,93]. While promising, this evidence remains preliminary and largely restricted to animal models.

In summary, all cell signaling processes involved in exercise adaptations are synthesized in Table 1.

## 4. Exercise-Induced Myokines and Inter-Organ Crosstalk

Regular physical exercise transforms skeletal muscle into a dynamic endocrine organ capable of secreting a wide array of bioactive molecules collectively known as myokines [105]. Produced in response to muscle contraction, these cytokine-like factors mediate autocrine and paracrine influences within the muscle and endocrine effects on distant organs [106,107]. Through these mechanisms, myokines act as molecular messengers linking physical activity to systemic metabolic regulation, immunomodulation, and energy homeostasis [39].

**Figure 2:**
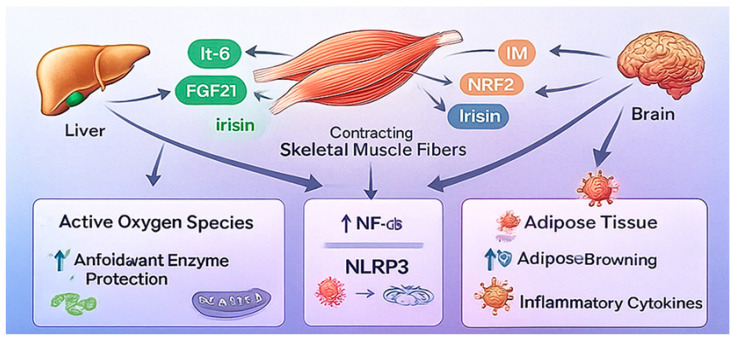
Exercise-Induced Myokines and Inter-Organ Crosstalk. Source: own elaboration.

A key challenge in the current literature is the heterogeneity in detection methods, variability in exercise protocols, and differences in population characteristics, which complicate the interpretation of myokine responses. Nonetheless, several consistent patterns have emerged and are summarized below (Figure 2).

### 4.1. Major Exercise-Induced Myokines

The best-characterized myokines—interleukin-6 (IL-6), irisin, myostatin, brain-derived neurotrophic factor (BDNF), fibroblast growth factor 21 (FGF21), and apelin—play central roles in mediating the multisystemic benefits of regular exercise [107,108].

IL-6, the prototypical myokine, can increase up to 100-fold during exercise, stimulating hepatic glucose output and adipose tissue lipolysis to match energetic demands [109]. Importantly, IL-6 also exerts anti-inflammatory effects by inducing IL-10 and IL-1 receptor antagonist and suppressing TNF-α signaling [110,111]. Its dual metabolic and immunomodulatory roles exemplify the context-dependent nature of myokines—acute versus chronic IL-6 signaling yields markedly different outcomes.

Irisin, generated through cleavage of the FNDC5 protein in a PGC-1α–dependent manner, is traditionally associated with endurance exercise [67,111,112]. It induces browning of white adipose tissue via UCP1 upregulation, increasing thermogenesis and energy expenditure, and may enhance insulin sensitivity and β-cell viability [113,114]. However, significant controversy persists regarding irisin quantification in humans, highlighting the need for improved methodological rigor.

Myostatin, a TGF-β family member, functions as a potent inhibitor of muscle growth. Exercise, particularly resistance training, suppresses myostatin, facilitating hypertrophy and metabolic improvements [115]. Reduced myostatin levels correlate with improved insulin sensitivity and lower adiposity, although human evidence remains less consistent than animal models [116]

BDNF is produced by both muscle and neural tissues and is induced via AMPK–PGC-1α mechanisms [117]. Locally, it enhances fatty acid oxidation, while systemically it supports neuroplasticity, cognition, and mood regulation, linking physical exercise with brain health [118,119].

FGF21, produced by muscle and liver, promotes fatty acid oxidation, glucose uptake, and hepatoprotection [120]. Apelin improves glucose uptake, vascular function, and AMPK activation, and its decline with age or metabolic disease suggests that exercise-induced restoration may counteract age-related metabolic deterioration [121,122].

Critically, although these myokines are frequently studied in isolation, their effects often overlap, and interactions among them remain poorly characterized.

### 4.2. Endocrine and Paracrine Signaling Mechanisms

Myokines shape metabolic and structural remodeling through autocrine/paracrine and endocrine routes. Locally, contraction-induced changes in AMP/ATP ratio, Ca^2+^ signaling, and ROS activate AMPK, MAPK, and PGC-1α pathways, thereby promoting mitochondrial biogenesis, angiogenesis, and fiber-type remodeling. Paracrine actions of IL-6, VEGF, and BDNF support oxidative metabolism, vascularization, and tissue repair [24,41,60,78].

Endocrinally, myokines circulate to distant organs, activating intracellular pathways such as JAK/STAT, PI3K/Akt, or p38 MAPK to influence lipid and glucose metabolism, inflammation, and tissue adaptation [123,124,125]. IL-6 regulates hepatic glucose production; apelin activates AMPK in adipose and vascular tissue; and irisin promotes adipose browning through integrin αV/β5 [126].

Beyond soluble proteins, extracellular vesicles and exosomes serve as carriers of myokines and myomiRs, protecting them from degradation and allowing tissue-specific delivery [127,128]. These exerkine-derived vesicles reveal that muscle communicates through a diverse molecular language extending beyond classical cytokines.

Critically, the relative contribution of vesicle-bound vs. free-circulating myokines is still unclear, and standardized isolation protocols are urgently needed.

### 4.3. Inter-Organ Communication: Muscle–Liver, Muscle–Pancreas, Muscle–Adipose, and Muscle–Brain Axes

Exercise-induced myokines coordinate a complex, multidirectional communication network linking skeletal muscle with the liver, pancreas, adipose tissue, and brain [17,41,129].

In the liver-muscle axis, IL-6 and FGF21 regulate hepatic glucose and lipid metabolism [109,126]. During acute exercise, IL-6 increases gluconeogenesis and glycogenolysis to maintain blood glucose [130,131]. With training, IL-6 promotes hepatic insulin sensitivity and lipid oxidation through AMPK and STAT3 activation [132,133]. FGF21 enhances fatty acid oxidation and reduces lipogenesis, offering protection against NAFLD [134].

In the muscle–pancreas axis, myokines influence β-cell survival and insulin secretion. Irisin enhances glucose-stimulated insulin release and β-cell viability [135,136]. IL-6 indirectly supports insulin secretion via GLP-1 induction. BDNF and apelin mitigate oxidative and inflammatory stress in β-cells, helping preserve endocrine function [112,117,137].

In the muscle–adipose axis, myokines regulate lipid metabolism and thermogenesis. Irisin, BAIBA, and METRNL promote browning via UCP1 upregulation, increasing energy expenditure and insulin sensitivity [138,139,140]. Exercise-induced suppression of myostatin enhances lipid mobilization and reduces adipose inflammation [141].

The muscle–brain axis, BDNF is a central mediator of muscle-to-brain signaling, promoting neurogenesis, synaptic plasticity, and cognitive benefits [106,118]. Irisin crosses the blood–brain barrier and stimulates hippocampal BDNF expression, amplifying neuroprotective effects [112,117,137]. Myokines such as apelin and cathepsin B further support neurovascular function and cognitive resilience [142].

Critically, many of these findings are derived from animal models or acute interventions; long-term human data remain limited.

### 4.4. Integrative Role of Myokines in Metabolism and Homeostasis

Exercise-induced myokines act as central regulators of whole-body metabolism, inflammation, and energy homeostasis, synchronizing adaptations across tissues [109,124,143]. Regular exercise promotes a myokine profile that enhances oxidative metabolism, insulin sensitivity, and anti-inflammatory signaling, whereas inactivity shifts this profile toward chronic low-grade inflammation [43,132,133,138].

Metabolically, IL-6, irisin, FGF21, and myonectin promote glucose uptake, lipid oxidation, and mitochondrial biogenesis. IL-6 exhibits dual roles: acutely promoting hepatic glucose output and chronically enhancing insulin sensitivity via AMPK and STAT3 [109,117,132,133]. Irisin stimulates adipose browning via UCP1, and FGF21 induces fasting-like adjustments including fatty acid oxidation and ketogenesis [112,134].

Immunometabolically, exercise shifts cytokine profiles toward anti-inflammatory states, mediated by IL-10, IL-1ra, and decreased TNF-α [109,110,111]. Apelin and BDNF support vascular and neuronal resilience, contributing to cardiometabolic and cognitive protection [112,117,137].

Myokines also influence liver lipid metabolism, β-cell survival, and adipose tissue remodeling [137]. Apelin and IL-15 improve β-cell mitochondrial function, while myostatin suppression promotes hypertrophy and reduces fat accumulation [135,136].

Disruption of myokine secretion—due to inactivity, obesity, aging, or chronic inflammation—leads to “myokine resistance,” a state of impaired tissue responsiveness that contributes to insulin resistance, sarcopenia, and cardiovascular disease [4,10,13,14]. Understanding these networks provides opportunities for therapeutic strategies such as exercise mimetics, recombinant myokines, and gene-based interventions [1,17,23,25,40].

Ultimately, myokines constitute a unifying framework linking mechanical activity to molecular health, reinforcing exercise as a systemic regulator of metabolic homeostasis [17,23,25,40]. The evidence presented here is synthesized in Table 2, which summarizes meta-analytic findings on key myokines and their responsiveness to exercise across diverse populations and modalities.

## 5. Translational and Clinical Perspectives: From Molecular Mechanisms to Therapeutic Applications

The elucidation of the molecular pathways activated by exercise has repositioned physical activity from a lifestyle recommendation to a potent therapeutic intervention for chronic non-communicable diseases (NCDs) [1,7,13]. As understanding of AMPK, PGC-1α, mTOR, myokines, and epigenetic regulators advances, the challenge lies in translating these mechanistic insights into clinically actionable strategies [1,17,23,25,40]. Bridging basic molecular biology with individualized, patient-centered interventions is central to the emergence of “exercise medicine”.

Clinically, structured aerobic and resistance training exert multimodal effects that target metabolic, cardiovascular, and neurodegenerative disorders [1]. Improvements in insulin sensitivity, reductions in systemic inflammation, enhanced mitochondrial biogenesis, and increased oxidative capacity have been consistently observed across tissues [17,21,58,115]. These adaptations mirror the pharmacological actions of therapies such as metformin (via AMPK activation), statins (via anti-inflammatory signaling), and neuroprotective agents that promote BDNF expression—supporting the conceptualization of exercise as a biological “polypill” with synergistic and pleiotropic benefits [150,151].

The concept of exercise mimetics—pharmacological agents that activate exercise-responsive pathways—represents another translational frontier. Compounds such as AICAR (AMPK agonist), resveratrol (SIRT1 activator), and GW501516 (PPARδ agonist) mimic aspects of endurance training by stimulating mitochondrial biogenesis and fatty acid oxidation [76,106,128,152]. Likewise, myostatin inhibitors aim to replicate the anabolic effects of resistance training. While promising, these agents only partially recapitulate the systemic benefits of exercise and raise concerns about safety, off-target effects, and ethical considerations. Future therapies will likely benefit from combined approaches in which pharmacological modulation complements, rather than replaces, physical activity to enhance metabolic and cellular resilience [153,154].

From a public health perspective, expanding knowledge of exercise-induced molecular mechanisms reinforces the imperative of incorporating structured physical activity into chronic disease prevention and management programs [1,17,23]. Early interventions that target muscle-derived signaling may prevent the development of metabolic inflexibility, systemic inflammation, and “myokine resistance”—a phenotype associated with sedentary behavior, obesity, and aging [40,41,110,114,125]. Understanding these mechanistic disruptions provides new avenues for mitigating the progressive decline in metabolic and functional capacity observed in high-risk populations.

**Figure 3:**
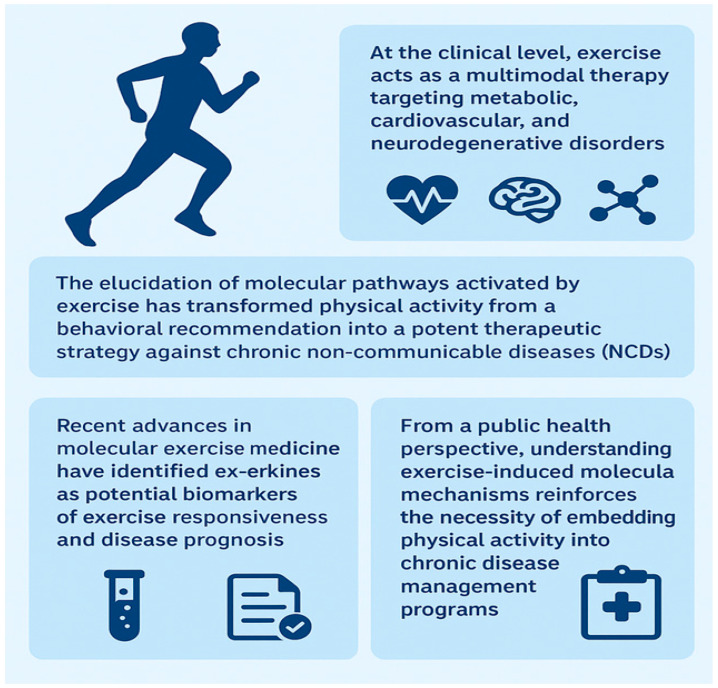
Translational and clinical perspectives from molecular mechanisms to therapeutic applications. Source: own elaboration.

In summary, deciphering the molecular underpinnings of exercise opens new avenues for translational and precision medicine. Integrating myokine biology, epigenetic adaptations, and exerkine profiling into clinical practice offers promising strategies to counter the growing burden of chronic diseases. By leveraging these mechanistic insights, exercise can be harnessed not only as a preventive measure but as a targeted therapeutic modality with substantial potential across metabolic, cardiovascular, and neurological domains [1] (Figure 3).

## 6. Limitations and Future Directions

Despite substantial progress in the molecular characterization of exercise-induced adaptations, several methodological and conceptual limitations continue to challenge the field. Heterogeneity in study designs, variability in training intensity and duration, and population differences (age, sex, metabolic status) hinder direct comparison across studies and make it difficult to establish universal mechanistic conclusions. Moreover, a considerable proportion of mechanistic insights derives from animal models or in vitro experiments, which only partially replicate the complex neuroendocrine, mechanical, and metabolic environment of human exercise.

A major limitation is the absence of standardized exercise protocols and sampling timelines. Acute and chronic exercise elicit distinct molecular signatures—yet many studies lack precise temporal profiling, leading to inconsistent interpretation of signaling cascades. This issue is particularly relevant for the quantification of circulating myokines, exerkines, and epigenetic markers, whose transient kinetics, low abundance, and sensitivity to external factors (diet, circadian rhythm, training status) complicate reliable measurement.

Advancing the field will require the integration of multi-omics approaches—including transcriptomics, proteomics, metabolomics, and epigenomics—within rigorously controlled human exercise trials. Such approaches can reveal tissue-specific, time-resolved adaptations and help reconcile inconsistencies observed in the current literature. However, implementing multi-omics designs in real-world exercise settings remains resource-intensive and logistically challenging, underscoring the need for methodological harmonization and accessible analytical pipelines.

Future research should prioritize translational and clinical frameworks that bridge mechanistic discoveries with personalized exercise prescriptions. Longitudinal studies incorporating molecular biomarkers, functional assessments, and clinical endpoints could clarify causal pathways, identify inter-individual variability in exercise responsiveness, and support precision exercise medicine. In this context, a key future direction is the development of personalized exercise prescriptions for individuals with metabolic disorders such as type 2 diabetes and obesity. Integrating molecular signatures—such as AMPK sensitivity, PGC-1α–driven mitochondrial remodeling, mTOR-related anabolic responses, and exercise-responsive microRNAs—could help tailor training modalities, intensities, and recovery strategies to individual metabolic profiles. Such mechanistically informed programs may optimize improvements in insulin sensitivity, lipid oxidation, and glycemic control, thereby enhancing clinical outcomes in these populations. Ultimately, progress in this field will depend on interdisciplinary collaboration among molecular biologists, clinicians, and exercise physiologists to develop standardized, reproducible, and clinically relevant models of exercise-induced molecular adaptation.

## 7. Conclusions

Exercise acts as a systemic modulator of molecular homeostasis, coordinating metabolic, inflammatory, and regenerative processes through tightly regulated signaling networks. Key pathways—including AMPK–PGC-1α, mTOR, MAPK, NF-κB, and epigenetic and miRNA-mediated regulation—form an integrated framework that translates mechanical and metabolic stress into adaptive responses across multiple organs.

Beyond its physiological benefits, exercise represents a biologically multifactorial and cost-effective therapeutic tool capable of preventing and mitigating chronic non-communicable diseases. By enhancing mitochondrial function, reducing inflammation, and optimizing energy balance, regular physical activity reprograms molecular networks toward resilience and longevity. Continued research into the molecular basis of exercise will not only refine our understanding of human adaptability but also open new therapeutic avenues for precision and translational medicine.

## Figures and Tables

**Table 1:** Summary of the main signaling pathways activated by exercise and their integrated molecular and physiological functions.

Ref.	Pathway	Primary Activators During Exercise	Key Molecular Targets	Molecular and Physiological Effects
[94,95]	AMPK	Increased AMP/ATP ratio, calcium flux and ROS	LKB1, CaMKKβ, ACC, CPT1, GLUT4, PGC-1α	Energy, oxidation and biogenesis
[61,96,97,98]	PGC-1α	AMPK, SIRT1, p38 MAPK Activation and endurance exercise	NRF1/2, ERRα, TFAM, VEGF	Mitochondria, oxidation and angiogenesis
[99,100]	mTOR	Mechanical overload, amino acids (leucine) and insulin/Akt signaling	S6K1, 4E-BP1, Raptor, PI3K/Akt	Synthesis, hypertrophy and anabolism
[80,83,101]	MAPK	Mechanical stress, cytokines and ROS	ERK1/2, JNK, p38 MAPK	Stress, cytokines and remodeling
[66,83,102,103]	NF-κB	ROS, cytokines (TNF-α, IL-1β) and metabolic stress	IKK complex, IκB degradation	Inflammation, redox and modulation
[87,89,90]	Epigenetic Regulation	Repeated muscle contraction and metabolic flux	DNA methyltransferases, histone acetyltransferases (HATs), SIRT1	Hypomethylation, acetylation and gene expression
[46,47,104]	microRNA	Muscle contraction, calcium signaling and oxidative stress	MyomiRs (miR-1, miR-133a/b, miR-206)	Myogenesis and mitochondria signaling

**Table 2:** Synthesis of Meta-Analytic Studies on Exercise-Responsive Myokines.

Author (Ref.)	Population	Exercise Type	Outcomes (95% CI)	Conclusion
Ringleb [110]	Healthy adults	Resistance	IL-6: 0.45 (0.29 to 0.61) IL-10: 0.14 (−0.09 to 0.36)	Acute inflammatory response
Jandová [144]	Healthy adults	Aerobics + resistance	Irisin: 0.39 (0.27 to 0.52)	Irisin increases
Bettariga [145]	Healthy adults	Aerobics + resistance	IL-15: 0.95 (−0.23 to 2.13) Irisin: 0.44 (−0.04 to 0.91) Secreted Acidic Protein and Rich in Cysteine 0.32 (−0.06 to 0.69) Oncostatin M: 0.08 (−2.40 to 2.56) Decorin: 0.99 (−11.14 to 13.12)	Evidence limited
Kazeminasab [146]	Adults	Aerobics and Anaerobic	Irisin overall: 0.15 (−0.35 to 0.65)	Irisin changes minimally
Vints [147]	Healthy adults	Chronic exercise	Neurotrophic factors: 0.427 (0.127–0.728) Pro-inflammatory factors: −0.013 (−0.316 to 0.290) Anti-inflammatory factors: 0.009 (−0.551–0.569) BDNF: 0.427 (0.127 to 0.728) Neurotrophin-3: 1.221 (0.213–2.228)	Exercise increases neurotrophins
Khalafi [148]	Healthy trained adults	Acute And chronic	Acute exercise IL-15: 0.90 (0.47 to 1.32) Chronic exercise IL-15: 0.002 (−0.51 to 0.51)	IL-15 shows variability
Torabi [149]	Adults with Overweight and obesity	Exercise Combined	Irisin: 0.957 (0.535–1.379)	Obesity modulates irisin

## Data Availability

No new data were created or analyzed in this study. Data sharing is not applicable to this article.
